# Supplementary material for: The development and psychometric properties of a self-report Catastrophizing Questionnaire
Source: R Soc Open Sci. 2021 Jan 13;8(1):201362. doi: 10.1098/rsos.201362 (PMC7890513; doi:10.1098/rsos.201362)
Supplement: Supplementary material [file rsos201362supp1.docx]

The development and psychometric properties of a self-report Catastrophising Questionnaire

# Pike, Alexandra C.*^1^, Serfaty, Jade*^1^, Robinson, Oliver J.^1^

# Supplementary introduction

Many constructs in psychiatry are closely related to each other, either causally or through as-yet-unknown mechanisms and predisposing factors. We believe catastrophising has this close-but-distinct relationship with other cognitive constructs. In particular, catastrophising can be distinguished from worry, defined as a prolonged period of rumination on a future concern or event (where rumination itself can be defined as excessively frequent thoughts about an event that has happened in the *past*). The content of worry does not necessarily have to be catastrophic – an individual may be worried about being late for the bus, or about their workload, without their concerns reaching the level of a perceived catastrophe. Equally, catastrophising does not have to entail worry, though it often will in practice (exemplifying their close relationship) – one can irrationally predict a catastrophe will occur without necessarily entering into a cycle of worry about it. The case where a worrying cycle features a catastrophe is referred to as ‘catastrophic worry’. This process, which involves worrying about something relatively minor which iteratively spirals into the prediction of a catastrophic event, is captured in the Catastrophising Interview Procedure (Vasey and Borkovec, 1992). Catastrophising is also related to poor perceived problem-solving ability, in so much as poor problem-solving skills may increase the likelihood of an individual engaging in catastrophising, because they do not believe that they are able to avert a catastrophe or that their actions in a difficult situation may, in fact, directly result in a catastrophe. Poor perceived problem-solving ability may form part of Beck’s construct of ‘minimisation’, which he pairs with ‘magnification’ and defines as *‘underestimation of the individual's performance, achievement or ability’* (Beck, 1963, p. 19). Additionally, research has shown that intolerance of uncertainty may precede catastrophic worry in Generalised Anxiety Disorder patients, and yet this relationship is not mediated by changes in sadness or anxiety (Meeten et al., 2012).

# Supplementary methods

## Procedure

In all studies, we asked participants if they had ever received a psychiatric diagnosis, and allowed them to respond yes or no. In Studies 1 and 2, we then asked whether they currently took any psychiatric medication, such as sertraline, citalopram, beta-blockers or diazepam.

From Study 3 onwards, we allowed participants to answer that they had taken psychiatric medication in the past but did not now. We also added two questions specific to depression/anxiety: we asked if they had been diagnosed with anxiety or depression (possible answers: yes/no), and whether they had ever taken medication specifically for anxiety or depression (possible answers: ‘Yes, I currently take medication’, ‘Yes, I took medication in the past’, and ‘No’).

## Quality assurance

We ensured data quality by removing participants who exceeded the recommended time allowed by Prolific (a calculation they base on the time we estimate it should take to complete all aspects of the study: the recommended time was over 30 minutes for all studies). In study 3 we also performed additional checks of data quality by manually examining the data of participants who responded repetitively, or showed divergent responses to items that were repeated. We did not perform these checks for earlier studies as these were preliminary studies to allow us to refine the questionnaire items. Additionally, it was only in study 3 that we included repeated items (in the short form and full form of the questionnaire), so we could only analyse divergence between these responses here.

## General analysis

### *Internal reliability and validity*

In order to evaluate the reliability of each scale, we examined two measures of internal consistency: Cronbach’s alpha, and Omega, which has been shown to be more robust than Cronbach’s alpha given assumption violations, and also has less risk of under- or over-estimating reliability (Dunn et al., 2014). Good values for Cronbach's alpha and Omega are above .7 or .8, with the caveat that this is not firm cut-off for acceptable reliability (Dunn et al., 2014). We also report mean inter-item correlations and mean item-total correlations (Table 1). Acceptable values of these means are around .40, indicating homogeneity, such that all items measure the same construct (catastrophising) and they do not all represent the same aspect of the construct (Field et al., 2012). Both the mean and distribution of inter-item correlations were examined as they are considered to provide a straightforward measure of internal consistency and homogeneity (Clark and Watson, 1995; Simms, 2008). The distribution of inter-item correlations should be moderate in magnitude and cluster around the mean value (Clark and Watson, 1995).

### *Exploratory factor analysis*

Exploratory factor analyses (EFA) using the minimum residual method were conducted in R (version 3.6.1) and the psych package (version 1.9.12.31) to examine the latent structure of the different versions of the questionnaire (Revelle, 2018). In order to verify whether our data was adequate for a factor analysis we looked at the Kaiser-Meyer-Olkin (KMO) measure of sampling adequacy, where we accepted values over .5 (Cerny and Kaiser, 1977), and Bartlett’s Test of Sphericity, a significant result in which indicates that the correlation matrix is significantly different from an identity matrix, so factor analysis is appropriate. For each study, to determine the number of factors, we considered several methods. Firstly, we ran a parallel analysis using the psych package. We also examined Kaiser’s eigenvalues, with the number of factors considered to be the number of eigenvalues greater than 1.0, and finally looked at the scree test even though it is thought to be less reliable than the first two methods (Zwick and Velicer, 1986). We used oblique rotation since we assumed the factors were correlated.

### *Confirmatory factor analysis*

Confirmatory factor analyses were run in lavaan, version 0.6-6. We considered several fit indices according to current conventions (Hu and Bentler, 1999; Worthington and Whittaker, 2006) and used the following cut-offs to indicate good fit: root mean square error of approximation (RMSEA) less than .08, standardized root mean square residual (RMSR) less than .08, Tucker-Lewis Index (TLI) close to .95, and comparative fit index (CFI) greater than .90.

### *Item reduction*

Item reduction was based on inter-item correlations, factor loadings, item uniqueness, and whether mental health clinicians (who we informally consulted during this process) thought an item captured elements of catastrophising. Items with a factor loading less than .35 were considered for removal, as well as items loading highly on more than one factor (Simms, 2008). Items with an inter-item correlation of less than .40 were also examined as they suggested that they were not highly related to the construct (Field et al., 2012). When two items had a high factor loading on the same factor and a high inter-item correlation, only one of them was retained. We also looked at the distribution of responses for each individual item in the aim of removing items that have highly skewed and unbalanced distributions (Clark and Watson, 1995). However, response distribution is dependent on the population tested, and it may be useful to have items that assess important information relevant to the construct in a specific population type even though the response distribution may be highly unbalanced. Therefore, we retained items that were endorsed highly by only a few individuals if clinicians agreed they were relevant to catastrophizing and might identify a more extreme level of catastrophizing symptoms (Clark and Watson, 1995). We also assessed item misfit using an Item Response Theory approach, implemented using the ‘mirt’ package in R (version 1.32.1), with a graded response model (for polytomous responses)(Samejima, 1997), assuming the same number of factors suggested by EFA. We classified items with poor fit as those with a significant value (*p* < .05) of the *S-X^2^* statistic (Orlando and Thissen, 2003), and either removed or modified these items. Items were rephrased when considered too complex, and removed if considered not to be specific to catastrophising (e.g. items focusing on “hopelessness”).

## Study 1: Development of an initial questionnaire

We first developed a 31 item-scale, items for which were generated from a) discussion with colleagues and clinicians, b) work published on pain catastrophising (Rosenstiel and Keefe, 1983; Sullivan et al., 2001) c) scales measuring dimensions that we thought were relevant to catastrophising such as the Barratt Impulsiveness Scale (Patton et al., 1995), the Catastrophising Subscale of the Coping Strategies Questionnaire (Rosenstiel and Keefe, 1983) and the Cognition Checklist (Beck et al., 1987). We also asked experienced clinically-trained colleagues to review the items we had included, who both added items and suggested the removal of others which were repetitive or not specific to catastrophising. Based on the discussion with colleagues, we theorised that for each individual, the level of catastrophising could be different according to the context (e.g. workplace, social life, health, relationships). Therefore, we included examples of catastrophising for each of these contexts. We pilot-tested the questionnaire within our research group prior to launching our main data collection efforts.

We also incorporated items targeting features of catastrophising which have been described in the literature, such as rumination, lack of confidence in problem solving, personal inadequacy and incompetence, a distorted perception of threat, magnification and lack of impulsivity (Geisser et al., 1994; Sullivan et al., 1995).

The items were phrased in two different ways, either naming features or symptoms of catastrophising such as “I turn minor issues into really big problems in my head” or using an example of catastrophising, “If I have a disagreement with my partner, I think that our relationship will end". In a few items including the latter example, we used “if” statements, as we conceptualise catastrophising to be based on rumination about possible future events. For the final scale see the Supplementary Materials, and for earlier iterations see the OSF page: [doi.org/10.17605/OSF.IO/CRFUW.](https://doi.org/10.17605/OSF.IO/CRFUW)

Participants were asked to rate each item on a five-point frequency-scale (never, rarely, sometimes, often, always) by deciding how often the statement has applied to them during the past two weeks. A two-week period was chosen to match other questionnaires that probe psychiatric symptoms (e.g. BDI-II, GAD-7), and because it is an empirical question as to whether catastrophising is stable over time as a ‘trait’ (Beck et al., 1996; Spitzer et al., 2006).

## Study 1 item reduction: creation of version 2 of the Catastrophising Questionnaire

Item reduction was performed according to the general methods section above. Six items showed a significant *S-X^2^* statistic, four of which we removed, and the remaining two were refined as they were considered to be very complex (with multiple sub-clauses). We removed other items by considering response distribution, low factor loadings and low inter-item correlations. All item response distributions are presented in the Supplement. We also removed items which loaded on the same factor and had a high inter-item correlation. For example, “If I have a disagreement with a person I care about, I think that I will lose that person” and “If I have a disagreement with my partner, I think that our relationship will end” both heavily loaded on factor 3 (.69 and .71 respectively) and had a strong inter-item correlation (.6). The choice between the two was made based on the idea that “a person I care about” was more general than “a partner” and was, therefore, applicable to a wider range of potential participants. This process reduced the scale from 31 items to 25 items, and all versions are available here: [doi.org/10.17605/OSF.IO/CRFUW.](https://doi.org/10.17605/OSF.IO/CRFUW)

## Study 2 item reduction: creation of version 3 of the Catastrophising Questionnaire

One item was removed from the second version of the questionnaire: “I overcomplicate my issues in my mind” since it was thought to be too complex and not specific to catastrophising. Additionally, this item had a significant *S-X^2^* value when we performed an IRT analysis, indicating that it did not fit the scale well. However, three other items (‘I think about all the ways that things can go wrong’, ‘If my partner is late home from work, I think that they have been in an accident’ and ‘I think that my house will be burgled’) also had significant *S-X^2^* values, but these were retained. The ‘burgled’ item was retained as we suspected that it might identify those with a more extreme level of catastrophising than was present in this sample - 117 individuals who were not selected for any psychopathology, none of whom rated ‘always’ for this item. The other two items were retained as they loaded highly onto the single factor, and showed good reliability and moderate relationships with other items.

## Study 2: procedure

Some participants received the items in a randomly shuffled order.

## Study 3: additional quality control checks

As well as the quality assurance methods used throughout all studies (which led to the removal of 35 participants in this study), we manually examined the data from a number of participants that we identified as potentially producing low-quality data. As we included a 4-item short version of our questionnaire which had an overlap of three items with the full scale, we were able to identify individuals who had high difference scores on the ratings of these items, indicating inconsistent responses. The maximum difference between the score of the three common items in the short form version and the full version of the questionnaire was 7 and the mean was 1.17. Because the possible maximum difference was 15 (the product of 3 items and maximal rating of 5), we considered there was no problematic difference in ratings. Additionally, we identified those who rated only one number (1, 3 or 5 only) for any questionnaire, which might indicate that they were not attending to the study. After manual examination of these data, no additional participants were removed.

## Study 3: Convergent and Discriminant measures

### *Convergent measures*

#### Anxiety

Catastrophising is known to relate to psychiatric diagnoses such as anxiety and depression, and as such should be related to scores on questionnaires measuring symptoms of these disorders. Therefore, we included the GAD-7 (Generalized Anxiety Disorder Assessment), which measures symptoms of generalized anxiety disorder over the previous two weeks (Spitzer et al., 2006). We also included the STAI-T (Trait Anxiety Subscale of the State Trait Anxiety Inventory) which measures anxiety level as an enduring personality trait, and focuses on anxiety in general rather than on a specific anxiety disorder (Spielberger et al., 1983).

#### Depression

For the same reasons as above, we included the PHQ-9 (Patient Health Questionnaire) which is a depression scale that asks about symptoms over the preceding two weeks (Spitzer et al., 1999). As we were testing participants online and could not assess risk of harm in person, we removed the question about suicidal ideation.

#### Worry

Worry is an important feature of anxiety disorders that may also be related to catastrophising. Thus, we included the PSWQ (Penn State Worry Questionnaire) which measures traits of worry independently from anxiety (Meyer et al., 1990). It examines the excessiveness, generality and uncontrollable dimensions of worry.

#### Rumination

Rumination is related to depression, and we thus hypothesised it would also correlate with catastrophizing. However, it is thought to be separable from depression, so in addition to the PHQ-9, we included the RRS (Rumination Response Scale) which measures two aspects: brooding and reflective pondering (Nolen-Hoeksma and Morrow, 1991).

### *Construct validity measures*

#### Cognitive Distortion Scale: Catastrophising Subscale

The Cognitive Distortion Scale (CDS) conceived by Covin et al. contains a 2-item Catastrophising subscale (Covin et al., 2011). This subscale was used for comparison with our Catastrophising Questionnaire to assess construct validity.

### *Discriminant measures*

#### Experience of Pleasure

We expected the experience of pleasure to be orthogonal to catastrophising as it implies a positive conception of the anticipation of pleasure and enjoying the present moment. Therefore, we included the TEPS-ANT and TEPS-CON (Temporal Experience of Pleasure Scale – Anticipatory and Consummatory subscales), which assess pleasure experienced during anticipation of reward and on attaining rewards, respectively (Gard et al., 2006).

## Study 4: Intra-class correlation for test-retest reliability

We assess test-retest reliability using an intraclass correlation coefficient. In particular, we used a two-way ICC, with absolute agreement (as we wanted a measure that is sensitive to mean differences between time 1 and time 2). This is known, in the terminology of McGraw and Wong (1996), as ICC(A,1). The effect of measure (the questionnaire) is assumed to be fixed, with random effects of participants. We also report the equivalent consistency ICC (C,1) (McGraw and Wong, 1996), which is identical to the Shrout and Fleiss, (1979) definition of an ICC(3,1). As the ICC tends to 0 it indicates low or no reliability, and as it tends to 1 it indicates increasingly perfect reliability. ICCs of below .4 indicate poor reliability, between .4 and .75 indicate moderate to good reliability, and above .75 can be interpreted as excellent reliability (Fleiss, 1999).

## Study 5: Discriminant measures

#### Alcohol Use

We expected alcohol use to show only a limited relationship with Catastrophising. We measured alcohol use using the Alcohol Use Disorder Identification Test (AUDIT) (Saunders et al., 1993). We tested discriminant validity using Spearman’s correlations, and HTMT values.

#### Schizotypy

We hypothesise that schizotypy and catastrophising are not highly related constructs, particularly above-and-beyond the effects of general psychiatric distress.

# Supplementary materials

All items from the final version of the Catastrophising Questionnaire, as used in Experiment 3, 4, and 5, are shown here. Participants were asked to indicate how often over the last two weeks the following statements had applied to them. They rate their responses on a scale with the following options: never, rarely, sometimes, often, always.

1. If I have a problem, I wish somebody else would take the burden away from me.
2. I think about all the ways that things can go wrong.
3. I imagine that I might have a serious health issue.
4. I think about things that others would say are unlikely to happen.
5. If I have an exam, I think that if I fail it will affect my whole future.
6. I think that we are facing a major environmental disaster that humankind will not survive.
7. I think that a disaster is going to happen to me.
8. If I have a disagreement with a person I care about, I think that we will not make up.
9. I overthink and then become unable to decide what to do.
10. I think I am going to make a big mistake soon.
11. If I have a medical symptom (headache, heart palpitations, stomach ache), I think I must have a serious disease.
12. If I have an illness, I don’t believe that treatment will work.
13. If I text a friend and they don’t message me back, I immediately think that they’re upset with me.
14. I think that any problem will only get worse as time passes.
15. If my partner is late home from work, I think that they have been in an accident.
16. I think that what I am going through is much worse than what others have experienced.
17. I think I am going to lose someone close to me forever.
18. I think that I will always have money problems.
19. I think that we will see another world war in the next few years.
20. If I have a bad month at work, I think that I will get fired.
21. I think that the worst case scenarios are very likely to happen.
22. I think that my house will be burgled.
23. I think about what will happen if I make a mistake.
24. I think that I am not very good at finding ways to solve my problems.

# Supplementary results

**Supplementary Table 1.** Sample characteristics for each study.

| **Demographic** | **Study 1** | **Study 2** | **Study 3** | **Study 4** | **Study 5** |
| --- | --- | --- | --- | --- | --- |
| **Mean age (sd)** | 30.0 (9.43) | 30.4 (8.29) | 29.9 (9.71) | 30.1 (9.92) | 31.3 (10.1) |
| **Females** | 50.4% | 61.5% | 48.2% | 47.0% | 47.3% |
| **Student status** | 36.8% | 33.3% | 41.0% | 33.0% | 37.5% |
| **UTC Timezone** |  | | | | |
| -7 | 3 | 7 | 7 | 1 | 4 |
| -6 | 3 | 0 | 4 | 1 | 2 |
| -5 | 8 | 13 | 26 | 2 | 9 |
| -4 | 13 | 14 | 40 | 3 | 18 |
| -3 | 2 | 0 | 3 | 1 | 2 |
| -2.5 | 0 | 1 | 1 | 0 | 0 |
| 0 | 0 | 1 | 2 | 0 | 2 |
| 1 | 47 | 47 | 245 | 35 | 127 |
| 2 | 27 | 27 | 136 | 47 | 83 |
| 3 | 13 | 6 | 38 | 9 | 14 |
| 8 | 0 | 0 | 0 | 0 | 1 |
| 9 | 1 | 1 | 0 | 1 | 0 |
| **Mental Illness** |  | | | | |
| Diagnosis (%) | 28 (23.9%) | 22 (18.8%) | 94 (18.8%) | 16 (16.0%) | 57 (21.6%) |
| Medication | 17 (14.5%) | 17 (14.5%) | - | - | - |
| Currently | *-* | *-* | 48 (9.6%) | 7 (7%) | 27 (10.2%) |
| In the past | *-* | *-* | 61 (12.2%) | 9 (9%) | 39 (14.7%) |
| **Anxiety/Depression** |  | | | | |
| Diagnosis | *-* | *-* | 176 (35.2%) | 34 (34%) | 53 (20.1%) |
| Medication |  | | | | |
| Currently | *-* | *-* | 51 (10.2%) | 7 (7%) | 27 (10.2%) |
| In the past | *-* | *-* | 103 (20.6%) | 22 (22%) | 36 (13.6%) |

Demographic information on participants from studies 1 to 5. We report mean and sd for the age of each sample, % female, % with student status, number in each timezone, number (and percentage of sample) who self-report a psychiatric diagnosis or current medication use for a psychiatric diagnosis.

**Supplementary Table 2**. Fit Indices for Exploratory Factor Analysis solutions.

| Scale version | Description | RMSR^[[1]](#footnote-1)^ | TLI^[[2]](#footnote-2)^ | RMSEA^[[3]](#footnote-3)^ |
| --- | --- | --- | --- | --- |
| Study 1 | EFA three-factor model | .05 | .91 | .05 |
| Study 2 | EFA one-factor model | .08 | .83 | .08 |
| Study 3 + convergent measures (97 items) | EFA five-factor model | .03 | .89 | .04 |

**Supplementary Table 3**. Means and standard deviations on the Catastrophising Questionnaire of all groups based on their psychiatric diagnoses and medication history.

| Question | **Yes** | | | | | | **No** | | |
| --- | --- | --- | --- | --- | --- | --- | --- | --- | --- |
|  | Mean | | | SD^[[4]](#footnote-4)^ | | n | Mean | SD |  |
| Psychiatric Diagnosis | 74.6 | | | 16.9 | | 94 | 58.1 | 16.9 | 406 |
| Diagnosis of anxiety or depression | 70.4 | | | 17.9 | | 176 | 56.2 | 16.2 | 324 |
|  | **Yes, currently** | | | **Yes, formerly** | | | **No** | | |
|  | Mean | SD | n | Mean | SD | n | Mean | SD | n |
| Taking  Psychiatric medication | 73.9 | 13.3 | 48 | 71.3 | 20.2 | 61 | 58.1 | 17.0 | 391 |
| Taking medication for anxiety or depression | 72.8 | 13.6 | 51 | 71.4 | 19.5 | 103 | 56.5 | 16.1 | 346 |

## Distributions of inter-item correlations for all three studies

### *Study 1*


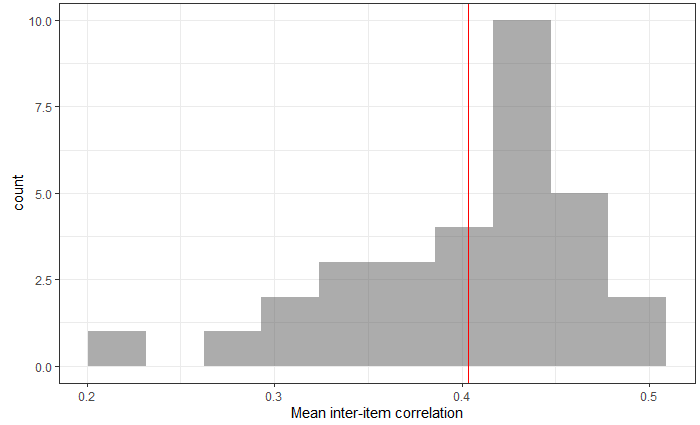


**Supplementary Figure 1:** Histogram of inter-item correlations for study 1. Red line indicates the mean inter-item correlation.

### *Study 2*


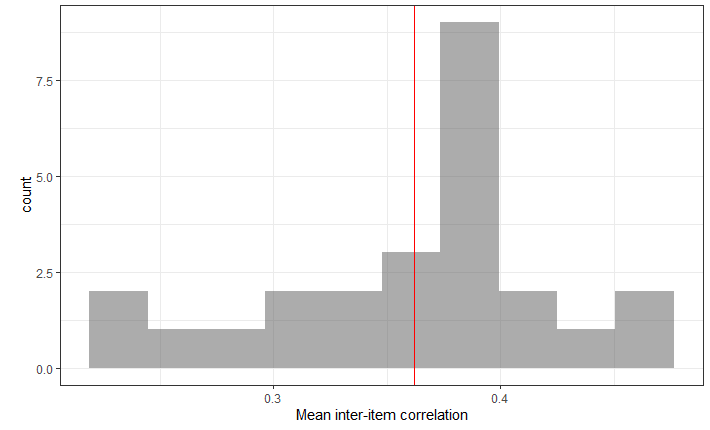


**Supplementary Figure 2:** Histogram of inter-item correlations for study 2. Red line indicates the mean inter-item correlation.

### *Study 3*


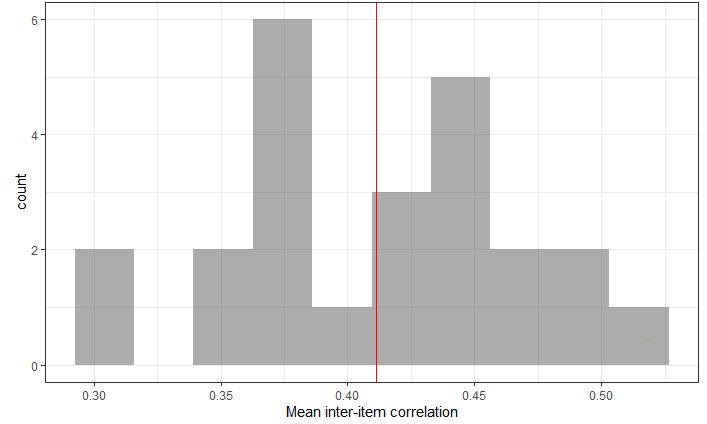


**Supplementary Figure 3:** Histogram of inter-item correlations for study 3. Red line indicates the mean inter-item correlation.

## Item response distributions for all three studies

### *Study 1*


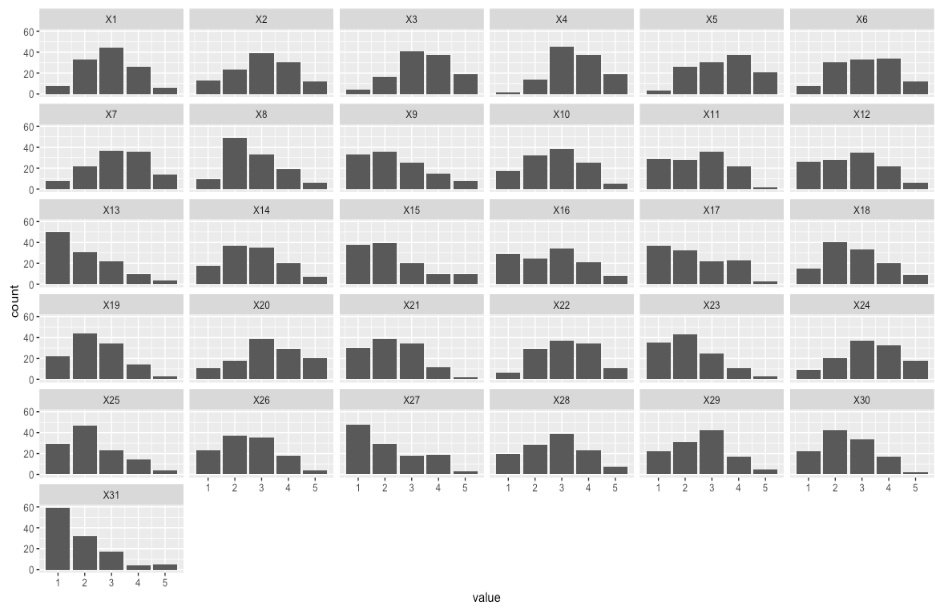


**Supplementary Figure 4:** Item response distributions for Study 1.

### *Study 2*


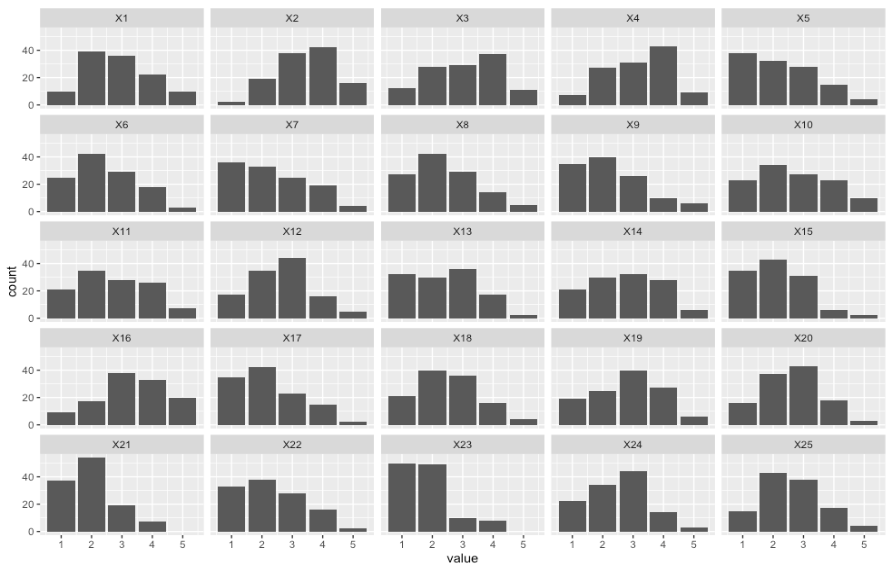


**Supplementary Figure 5:** Item response distributions for Study 2.

### *Study 3*


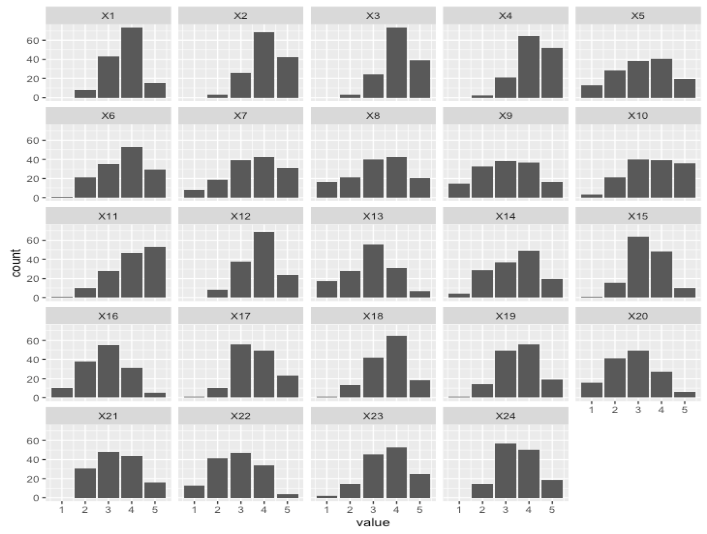


**Supplementary Figure 6:** Item response distributions for Study 3.

## Structural Equation Modelling

We performed a confirmatory factor analysis within a Structural Equation Modelling framework to identify the relationships between the different constructs from Study 3 that we consider ‘convergent’. Notably all the correlations are very high, but this is not exclusive to the ‘catastrophising’ latent variable. This may reflect an underlying ‘psychological distress’ or ‘p’ factor.
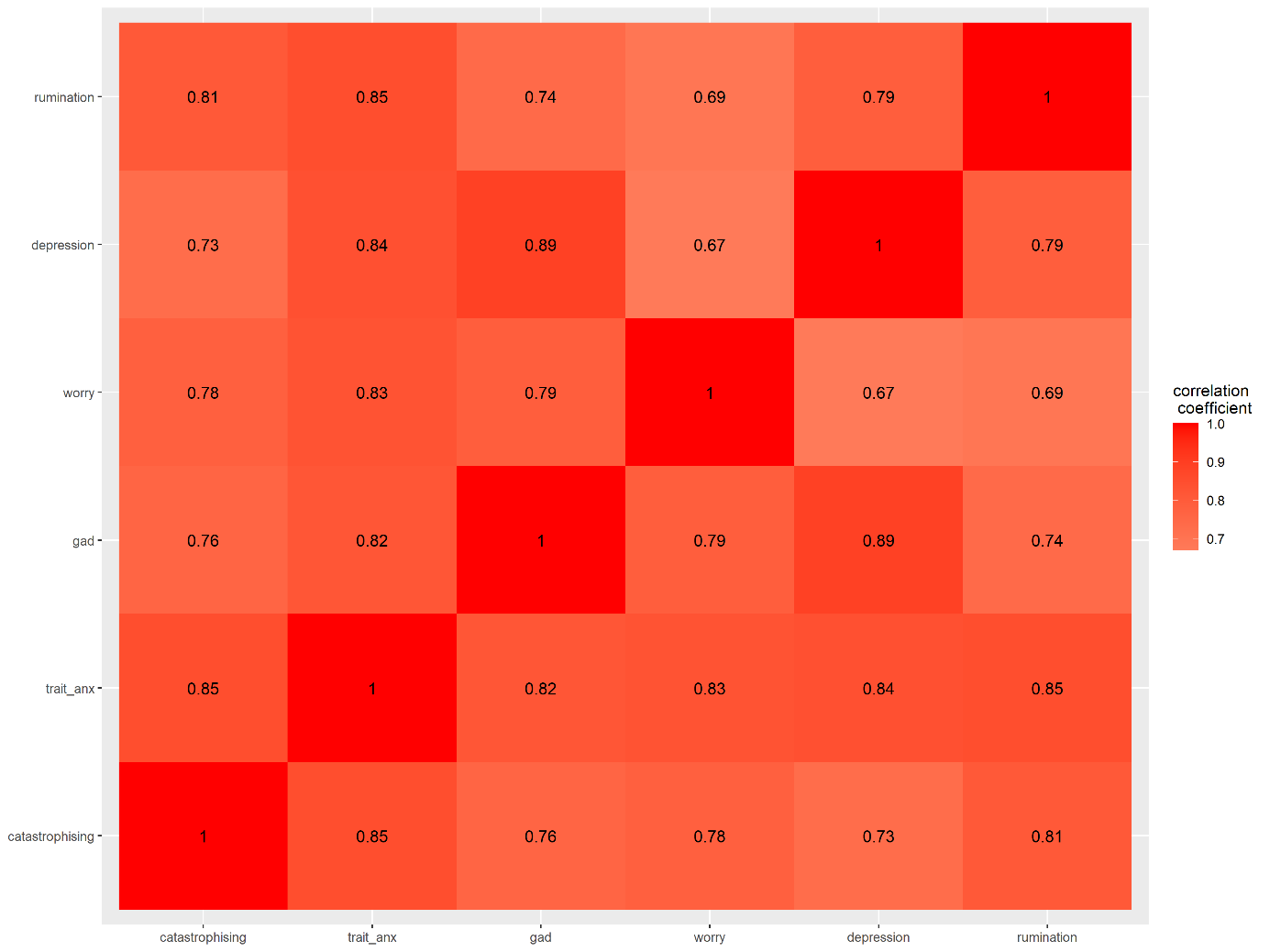


**Supplementary Figure 7:** Correlation plot displaying the correlations between latent factors estimated using a Confirmatory Factor Analysis in an SEM framework for all convergent measures from study 3.

## Correlations between scales used in Study 5
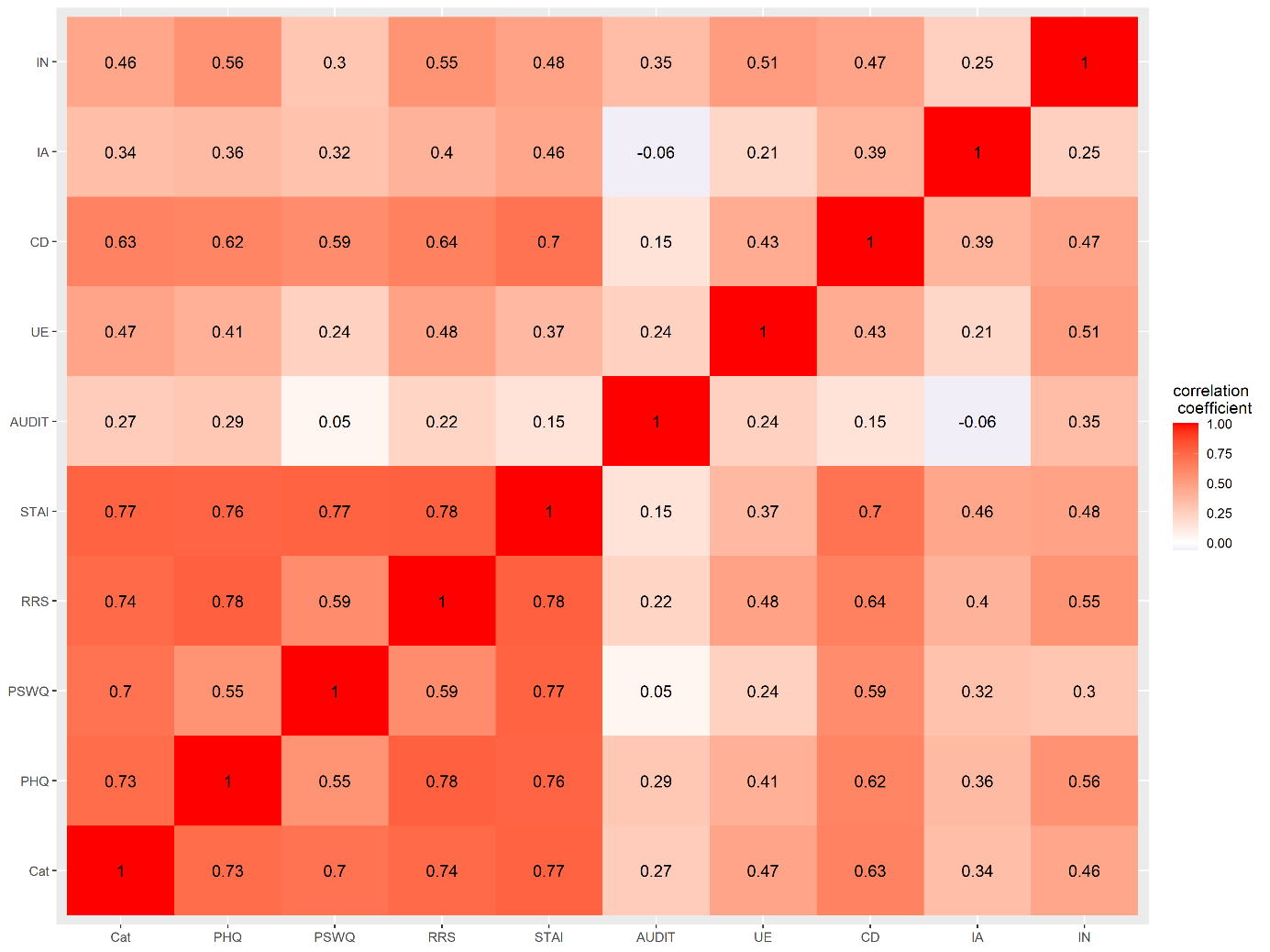


**Supplementary Figure 8:** Correlation plot displaying the correlations between the Catastrophising Questionnaire and all scales used for discriminant and convergent measures in Study 3.

## Supplementary references

Beck, A.T., 1963. Thinking and Depression: I. Idiosyncratic Content and Cognitive Distortions. Arch Gen Psychiatry 9, 324. https://doi.org/10.1001/archpsyc.1963.01720160014002

Beck, A.T., Brown, G., Steer, R.A., Eidelson, J.I., Riskind, J.H., 1987. Differentiating anxiety and depression: A test of the cognitive content-specificity hypothesis. Journal of Abnormal Psychology 96, 179–183. https://doi.org/10.1037/0021-843X.96.3.179

Beck, A.T., Steer, R.A., Ball, R., Ranieri, W.F., 1996. Comparison of Beck Depression Inventories-IA and-II in Psychiatric Outpatients. Journal of Personality Assessment 67, 588–597. https://doi.org/10.1207/s15327752jpa6703_13

Cerny, B.A., Kaiser, H.F., 1977. A Study Of A Measure Of Sampling Adequacy For Factor-Analytic Correlation Matrices. Multivariate Behavioral Research 12, 43–47. https://doi.org/10.1207/s15327906mbr1201_3

Clark, L.A., Watson, D., 1995. Constructing validity: Basic issues in objective scale development. Psychological Assessment 7, 309–319. https://doi.org/10.1037/1040-3590.7.3.309

Dunn, T.J., Baguley, T., Brunsden, V., 2014. From alpha to omega: A practical solution to the pervasive problem of internal consistency estimation. Br J Psychol 105, 399–412. https://doi.org/10.1111/bjop.12046

Field, A., Miles, J., Field, Z., 2012. Discovering Statistics Using R. SAGE Publication Ltd.

Fleiss, J.L., 1999. The Design and Analysis of Clinical Experiments. John Wiley & Sons, Inc., Hoboken, NJ, USA. https://doi.org/10.1002/9781118032923

Geisser, M.E., Robinson, M.E., Keefe, F.J., Weiner, M.L., 1994. Catastrophizing, depression and the sensory, affective and evaluative aspects of chronic pain: Pain 59, 79–83. https://doi.org/10.1016/0304-3959(94)90050-7

Hu, L., Bentler, P.M., 1999. Cutoff criteria for fit indexes in covariance structure analysis: Conventional criteria versus new alternatives. Structural Equation Modeling: A Multidisciplinary Journal 6, 1–55. https://doi.org/10.1080/10705519909540118

McGraw, K.O., Wong, S.P., 1996. Forming inferences about some intraclass correlation coefficients. Psychological Methods 1, 30–46. https://doi.org/10.1037/1082-989X.1.1.30

Meeten, F., Dash, S.R., Scarlet, A.L.S., Davey, G.C.L., 2012. Investigating the effect of intolerance of uncertainty on catastrophic worrying and mood. Behaviour Research and Therapy 50, 690–698. https://doi.org/10.1016/j.brat.2012.08.003

Orlando, M., Thissen, D., 2003. Further Investigation of the Performance of S - X2: An Item Fit Index for Use With Dichotomous Item Response Theory Models. Applied Psychological Measurement 27, 289–298. https://doi.org/10.1177/0146621603027004004

Patton, J.H., Stanford, M.S., Barratt, E.S., 1995. Factor structure of the Barratt impulsiveness scale. J Clin Psychol 51, 768–774. https://doi.org/10.1002/1097-4679(199511)51:6<768::aid-jclp2270510607>3.0.co;2-1

Revelle, W., 2018. psych: Procedures for Personality and Psychological Research. Northwestern University, Evanston, Illinois, USA.

Rosenstiel, A.K., Keefe, F.J., 1983. The use of coping strategies in chronic low back pain patients: Relationship to patient characteristics and current adjustment: Pain 17, 33–44. https://doi.org/10.1016/0304-3959(83)90125-2

Samejima, F., 1997. Graded Response Model, in: van der Linden, W.J., Hambleton, R.K. (Eds.), Handbook of Modern Item Response Theory. Springer New York, New York, NY, pp. 85–100. https://doi.org/10.1007/978-1-4757-2691-6_5

Saunders, J.B., Aasland, O.G., Babor, T.F., De La Fuente, J.R., Grant, M., 1993. Development of the Alcohol Use Disorders Identification Test (AUDIT): WHO Collaborative Project on Early Detection of Persons with Harmful Alcohol Consumption-II. Addiction 88, 791–804. https://doi.org/10.1111/j.1360-0443.1993.tb02093.x

Shrout, P.E., Fleiss, J.L., 1979. Intraclass correlations: Uses in assessing rater reliability. Psychological Bulletin 86, 420–428. https://doi.org/10.1037/0033-2909.86.2.420

Simms, L.J., 2008. Classical and Modern Methods of Psychological Scale Construction. Social Pers Psych Compass 2, 414–433. https://doi.org/10.1111/j.1751-9004.2007.00044.x

Spitzer, R.L., Kroenke, K., Williams, J.B.W., Löwe, B., 2006. A Brief Measure for Assessing Generalized Anxiety Disorder: The GAD-7. Archives of Internal Medicine 166, 1092. https://doi.org/10.1001/archinte.166.10.1092

Sullivan, M.J.L., Bishop, S.R., Pivik, J., 1995. The Pain Catastrophizing Scale: Development and validation. Psychological Assessment 7, 524–532. https://doi.org/10.1037/1040-3590.7.4.524

Sullivan, M.J.L., Thorn, B., Haythornthwaite, J.A., Keefe, F., Martin, M., Bradley, L.A., Lefebvre, J.C., 2001. Theoretical Perspectives on the Relation Between Catastrophizing and Pain: The Clinical Journal of Pain 17, 52–64. https://doi.org/10.1097/00002508-200103000-00008

Vasey, M.W., Borkovec, T.D., 1992. A catastrophizing assessment of worrisome thoughts. Cogn Ther Res 16, 505–520. https://doi.org/10.1007/BF01175138

Worthington, R.L., Whittaker, T.A., 2006. Scale Development Research: A Content Analysis and Recommendations for Best Practices. The Counseling Psychologist 34, 806–838. https://doi.org/10.1177/0011000006288127

Zwick, W.R., Velicer, W.F., 1986. Comparison of five rules for determining the number of components to retain. Psychological Bulletin 99, 432–442. https://doi.org/10.1037/0033-2909.99.3.432

1. Root Mean Square of the Residuals (good fit indicated by < .08) [↑](#footnote-ref-1)
2. Tucker-Lewis Index (good fit indicated by close to .95) [↑](#footnote-ref-2)
3. Root Mean Square Error of Approximation (good fit indicated by <.08) [↑](#footnote-ref-3)
4. Standard deviation [↑](#footnote-ref-4)
